# Bridging the human-AI gap: the role of perceived teacher support in shaping EFL learners’ willingness to engage in collaborative writing

**DOI:** 10.3389/fpsyg.2026.1822545

**Published:** 2026-06-18

**Authors:** Wenting Gong, Chuang Xu, Jian-Hong Ye

**Affiliations:** 1 School of Languages and Cultures, Hunan Institute of Technology, Hengyang, Hunan, China; 2 School of Marxism, Hunan Institute of Technology, Hengyang, Hunan, China; 3 Faculty of Education, Beijing Normal University, Beijing, China

**Keywords:** perceived teacher support, willingness to engage, human-AI collaborative writing, task flow, technology acceptance, Stimuli-Organism-Response (S-O-R) framework, L2 writing, Chinese EFL learners

## Abstract

Collaborative writing instruction has been widely adopted in second language (L2) writing pedagogy, and the human-AI collaborative approach has recently gained increasing traction in this field. Despite the promising potential of this pedagogical model in facilitating L2 writing, the factors influencing learners’ willingness to engage (WTE) in human-AI collaborative L2 writing remain underexplored. Based on the Stimulus-Organism-Response (S-O-R) model and the Technology Acceptance Model (TAM), this study explores how perceived teacher support contributes to EFL learners’ WTE in human-AI collaborative L2 writing. A total of 503 Chinese university EFL learners with AI-aided L2 writing experience were included in this quantitative study. Findings from questionnaires and regression analyses indicate that perceived teacher support is directly and positively linked to the EFL learners’ WTE. It also exerts an indirect association with WTE via the independent mediating roles of technology acceptance and task flow. Furthermore, technology acceptance and task flow jointly form a chain-mediating pathway between perceived teacher support and WTE. These findings not only broaden the application of the S-O-R model and TAM within human-AI collaborative writing contexts but also provide valuable implications for L2 writing pedagogy in AI-aided contexts.

## 1 Introduction

As a cornerstone of English as a foreign language (EFL) development, second language (L2) writing is indispensable for fostering learners’ overall language proficiency, practical application ability, meta-cognitive skills, and higher-order cognitive competencies (Farsani et al., 2025; Luo et al., 2025), thereby supporting their academic development (Zou et al., 2024). Compared with other language skills such as reading and speaking, L2 writing presents substantial challenges for EFL learners owing to their limited linguistic resources and intense cognitive load, which may consequently increase their level of writing anxiety (Cheng, 2004). For university students, argumentative writing represents the most prevalent genre in L2 writing instruction, not only because it dominates L2 assessment tasks but also because it effectively cultivates critical thinking skills (Pratama et al., 2025; Zou et al., 2024). Collaborative writing has long been recognized as a fundamental pedagogical approach that enhances L2 writing outcomes, promotes EFL learners’ cognitive development, and fosters their cooperative skills in L2 writing contexts (Elabdali, 2021; Li and Zhang, 2023; Storch, 2011). The recent surge of artificial intelligence (AI) technologies has spurred a revolutionary change, making human-AI collaborative writing an inevitable trend within higher education and language learning ecosystems. This newly emerged symbiotic relationship between humans and AI in L2 writing typifies the integration of intelligent systems into human agency (Dhillon et al., 2024). Recent empirical evidence indicates that human-AI collaborative writing in L2 classrooms can significantly improve EFL learners’ writing efficiency and overall text quality (Yan, 2024; Yang et al., 2025).

Students’ willingness to engage (WTE), conceptualized as the dynamic antecedent state that comes before actual engagement and is related to cognitive, behavioral, motivational, and affective aspects (Lin and Wang, 2025; Wang and Mercer, 2020), functions as the crucial prerequisite for maintaining collaborative learning efforts (Weinberger and Shonfeld, 2020). For EFL learners, various contextual and individual factors may relate to their WTE in L2 writing, especially when completing writing tasks with AI tools, which require additional technical and emotional adjustment beyond traditional writing. These influencing factors include characteristics of the task, the level of social support, the learner’s physical and emotional conditions (Kim, 2020; Wang and Mercer, 2020). The interplay of these variables carries particular significance in technology-aided L2 writing contexts, which demand active cognitive and affective investment. Within such environments, learners’ technology acceptance shapes their readiness to utilize AI tools, while their task flow experience generates positive affective states and further enhances EFL learners’ learning motivation (Li et al., 2026). At the same time, educators face restructured scaffolding responsibilities that require pedagogical practices addressing both language development and the cultivation of digital literacy (Deng et al., 2026; Dhillon et al., 2024).

While an existing body of literature has affirmed the crucial role of teachers in EFL learning success (Gong and Xu, 2024; Yu et al., 2024), their support perceived by EFL learners in AI-aided L2 writing contexts within higher education remains under-explored. This research gap suggests that the instructional framework shaped by teacher support and the technological framework determined by AI tool characteristics differ significantly from those in traditional L2 writing contexts, which may consequently shape students’ willingness to engage in such tasks. These external environmental stimuli may interact with learners’ individual factors, such as technology acceptance and flow experience, to collectively shape their willingness to engage in Human-AI collaborative L2 writing. Such complex interactions underscore the necessity of adopting an integrated theoretical lens that combines the Stimulus-Organism-Response (S-O-R) framework with Technology Acceptance Model (TAM) principles. Based on the integrated theoretic framework, this study aims to address two research questions:

(1) How does perceived teacher support contribute to EFL learners’ willingness to engage in human-AI collaborative L2 writing?
(2) How do technological perception (technology acceptance) and learners’ psychological state (task flow) mediate the relationship between perceived teacher support and willingness to engage in human-AI collaborative L2 writing?

## 2 Theoretical framework and research hypothesis

### 2.1 Integrated theoretical framework of S-O-R and TMA

The S-O-R (Stimulus-Organism-Response) is a theoretical framework proposed from the perspective of environmental psychology to explicate the psychological decision-making process of individuals. This theoretical model posits that environmental factors, functioning as external stimuli (S), exert influence on an individual’s cognitive or affective states (O), thereby inducing modifications in their behavioral responses (R) (Mehrabian and Russell, 1974). In recent academic applications, this paradigm has been extended to explore learners’ learning behaviors and performance in AI-aided educational settings (e.g., Liu et al., 2025). In human-AI collaborative L2 writing instruction, EFL learners rely on AI tools rather than direct peer collaboration to compose L2 essays. Therefore, technological environment and characteristics of teacher support differ from those in traditional L2 writing classrooms, which may act as new stimuli that shape learners’ internal affective and cognitive experiences.

The Technology Acceptance Model (TAM), as a groundbreaking theoretical framework within information systems, simulates individuals’ decision-making processes in adopting technological innovations. At the core of this theory lies the idea that external environmental factors in technological systems affect users’ attitudinal and behavioral reactions via two beliefs: perceived usefulness and perceived ease of use (Davis, 1989; Venkatesh and Davis, 2000). In the field of L2 writing research, this model has been effectively applied to examine students’ acceptance of diverse educational technologies, such as automated English writing evaluation systems and generative AI tools (Liang et al., 2026; Zhai and Ma, 2022). Empirical findings indicate that technology acceptance is significantly linked to learners’ motivational levels, affective states, and behavioral engagement (Guo and Wang, 2025; Huang et al., 2024). Such evidence validates the applicability of the TAM in technology-mediated language education and provides empirical support for technology-aided foreign language pedagogy.

**FIGURE 1:**
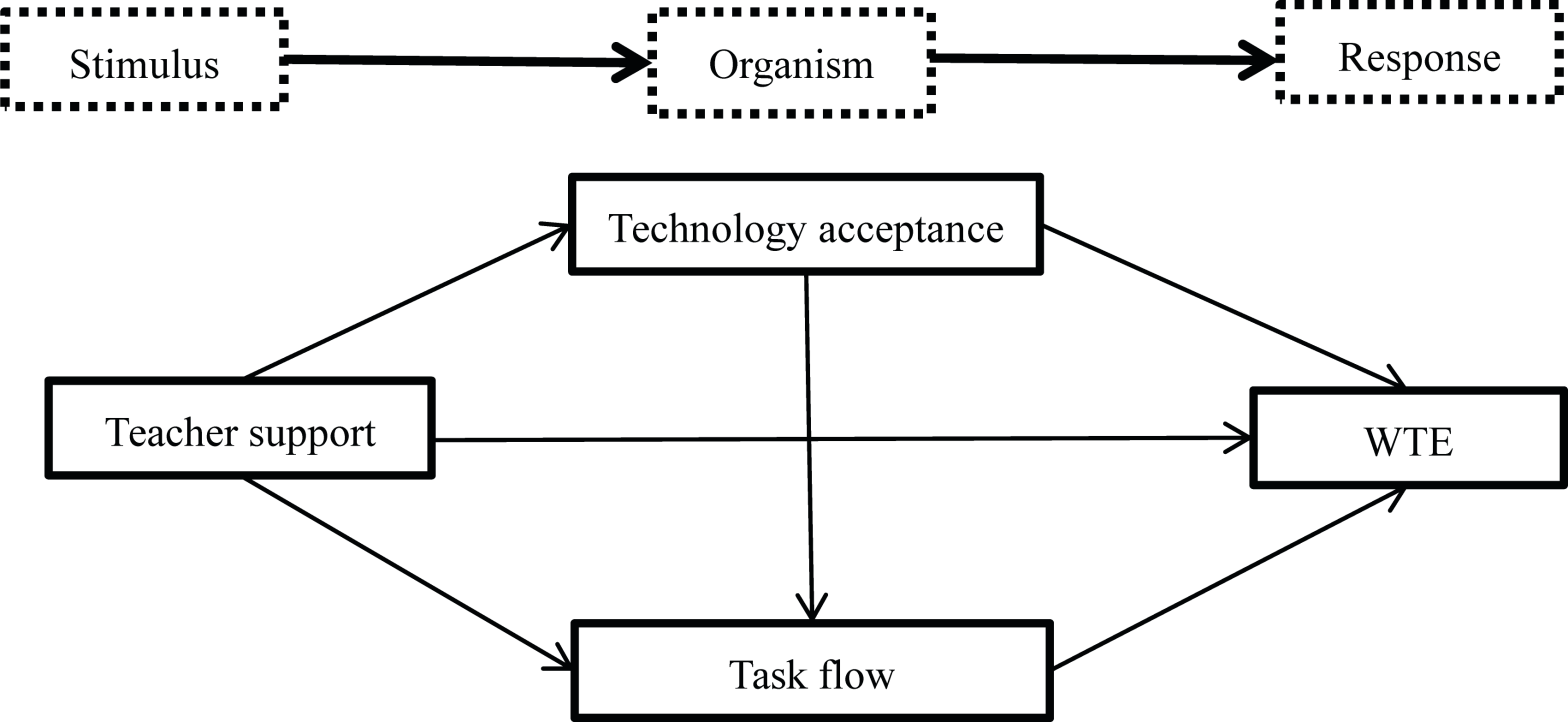
The theoretical framework.

Scholars frequently integrate TAM with other behavioral theories to analyze complex cognitive and behavioral mechanisms (Fareed and Kirkil, 2025). This study proposes an integrated theoretical framework by combining the TAM with the S-O-R paradigm, aiming to clarify both external and internal factors that shape EFL learners’ willingness to engage in human-AI collaborative L2 writing tasks. In this theoretical framework, perceived teacher support is conceptualized as an external environmental stimulus, representing social-instructional inputs that learners perceive from teachers in AI-aided contexts. Technology acceptance is construed as a rational cognitive appraisal process through which learners evaluate the utility and ease of using AI tools, whereas task flow experience represents a profound psychological state marked by intense focus, full immersion, and intrinsic enjoyment during task completion. Together, these two variables constitute internal organismic mediators that translate external environmental stimuli into learners’ subsequent willingness to engage (WTE) in human-AI collaborative L2 writing tasks, which serves as a behavioral response variable, indicating decision-making outcomes in AI-aided learning contexts, as illustrated in Figure 1.

### 2.2 Perceived teacher support and EFL learners’ WTE in human-AI collaborative writing

Teacher support denotes a set of supportive resources offered by teachers. These resources cover emotional, cognitive, behavioral, and instrumental dimensions, aiming to enhance students’ academic performance and psychological wellbeing (Ding and Wang, 2025; Huang et al., 2024). It holds particular significance in EFL learning contexts given that L2 acquisition inherently involves complex and challenging processes of cognitive restructuring, cultural adaptation, and identity exploration (Dewaele and Li, 2020; Zhang, 2019). As a critical gauge of overall language proficiency, L2 writing has long been recognized as a major hurdle for EFL learners (Klimova, 2014; Zhang and Zhang, 2024). The advent of collaborative writing pedagogy has introduced a new paradigm for L2 writing instruction in tertiary institutions. Findings suggest that through collaborative mechanisms, L2 learners can effectively enhance linguistic accuracy, textual coherence, and meta-cognitive abilities (Storch, 2011). The effective implementation of such educational approaches highly depends on the professional assistance of teachers. For example, teachers should employ pedagogical techniques to promote deep learning (Storch, 2013), and create psychologically safe environments to reduce EFL learners’ L2 writing anxiety (Zhang and Jiang, 2025). As AI technologies are deeply incorporated into the L2 writing process, the human-AI collaborative writing model brings new requirements for EFL learners’ ability to integrate technology (Stan, 2022). Therefore, teacher support becomes even more essential, as it enables learners to acquire technological skills and linguistic knowledge that would otherwise be hard to achieve on their own (Dhillon et al., 2024), which in turn relates to their willingness to engage in human-AI collaborative L2 writing. Although perceived teacher support appears to be a product of individual psychological processing, it is directly shaped by actual teacher support behaviors. Accordingly, perceived teacher support is regarded as a valid and critical indicator for measuring teacher support and has been widely adopted in numerous educational studies (Gong and Xu, 2024; Yang and Du, 2023). Empirical studies also validates that perceived teacher support plays a crucial role in enhancing EFL learners’ motivation and engagement within technology-mediated learning environments (Yang and Du, 2023; Yu et al., 2024). Hence, this study puts forward Hypothesis 1:

Perceived teacher support is positively associated with EFL learners’ willingness to engage in human-AI collaborative L2 writing.

### 2.3 The mediating role of technology acceptance

Technology acceptance, conceptualized through the Technology Acceptance Model (TAM) (Davis, 1989), reflects users’ attitudes regarding the utilization of a specific technology. At its core, TAM puts forward two mutually-reliant elements: perceived usefulness (PU) and perceived ease of use (PEOU) (Davis, 1989). In educational contexts, this interdependence manifests as a cyclic relationship where enhanced PEOU amplifies PU by lessening cognitive hurdles in adopting technology (Wang et al., 2025).

Teacher support severs as a critical external variable shaping EFL learners’ technological perceptions. Through scaffolding methods such as explicit instructions, guided interactions, and timely feedback, educators help learners form their initial technological mental models and later restructure their cognition. This in turn stimulates learners’ internal motivation and ability to learn with AI technologies (Chiu et al., 2024). Previous research also highlights the critical role of structured teacher guidance in fostering students’ readiness to integrate AI tools into L2 writing practices (e.g., Deng et al., 2026). Additionally, the acceptance of technology is reciprocally associated with their internal motivation when incorporating technologies into learning. Studies reveal that positive attitudes toward educational technologies are linked to engagement behaviors via competence development and autonomy satisfaction (Huang et al., 2024). Within the context of higher education, the extent to which students embrace technologies like AI tools can effectively forecast their intentions to adopt these technologies and their actual levels of involvement (Cai, 2025; Guo and Wang, 2025). Regarding L2 writing specifically, perceived usefulness and perceived ease of use have been validated as key correlates of Chinese EFL learners’ attitudes toward generative AI tools, which further link to their behavioral intention to adopt such tools as English writing assistants (Liang et al., 2026). Moreover, the mediating role of technology acceptance between contextual factors and behavioral intentions has been examined in recent studies (Chahal and Rani, 2022; Liang et al., 2026). Based on this, the current study puts forward Hypothesis 2:

Technology acceptance mediates the relationship between perceived teacher support and EFL learners’ willingness to engage in human-AI collaborative L2 writing.

### 2.4 The mediating role of task flow

Flow experience is a psychological state characterized by complete cognitive immersion and enhanced intrinsic motivation during goal-directed activities (Csikszentmihalyi et al., 2014). An optimal emotional state brought about by a particular task is what’s known as task flow (Czimmermann and Piniel, 2016), and it has been empirically proven to be related to various aspects of positive emotions within learning settings. Among these are enhanced wellbeing, sustained task enjoyment, and increased satisfaction with learning outcomes (Egbert, 2003; Hwang et al., 2025). Within tertiary education settings, emerging evidence substantiates the existence of flow states during collaborative L2 writing tasks (Liu and Song, 2021; Zuniga and Payant, 2021). The challenge-skills balance hypothesis lies at the core of flow theory. This hypothesis suggests that when the perceived difficulties of tasks match the self-evaluated capabilities of learners, the most favorable psychological involvement takes place (Csikszentmihalyi et al., 2014; Czimmermann and Piniel, 2016). In the practice of human-AI collaborative L2 writing tasks characterized by heightened cognitive-linguistic demands, strategic scaffolding from teachers may bridge competence gaps through targeted linguistic support and foster perceived controllability over complex writing tasks (Fan et al., 2024). This, in turn, provides favorable conditions for EFL learners’ flow experience to emerge. The contributing role of teacher support in shaping EFL learners’ flow has been documented in studies across multiple contexts (Dewaele et al., 2022; Gong and Xu, 2024).

As an optimal psychological state, flow experience functions as a self-reinforcing mechanism that stimulates intrinsic motivation cycles and sustains task engagement (Li et al., 2026). Within the L2 writing contexts aided by AI technology, task flow experience has been demonstrated to promote learners’ willingness to engage through internalized learning trajectories (Lv et al., 2024). Recent empirical research has also established the mediating role of flow experience in converting instructional support into individuals’ willingness to engage in sustained learning (Huang et al., 2025). Therefore, this study puts forward Hypothesis 3:

Task flow experience mediates the relationship between perceived teacher support and EFL learners’ willingness to engage in human-AI collaborative L2 writing.

### 2.5 The chained mediation of technology acceptance and task flow experience

According to TAM, individuals have limited attentional and effort resources, and technology can facilitate the optimal allocation of these resources to achieve more efficient task performance (Davis, 1989). Therefore, those who exhibit a greater acceptance of technology are more likely to possess flexible thinking, gain access to a broader array of resources, and demonstrate a stronger ability to adapt to alterations in the technology-aided learning environment. As a result, learners’ sense of academic self-efficacy is enhanced. Flow experience in online learning contexts has been shown to have academic self-efficacy as one of its significant precursors (Hong et al., 2025). In addition, collaborative activities are more conducive to the occurrence of task flow compared to solitary ones (Dewaele et al., 2022). Also, recent developments in flow research highlight the interactive relationship between task-related features and learner agency in L2 learning contexts, which may create optimal conditions for the emergence of flow (Li et al., 2026). Against this backdrop, when students hold positive attitudes toward engaging in human-AI collaborative writing tasks, they may enter a state of flow more easily than in traditional pen-and-paper writing. Empirical research has also confirmed the association between technology acceptance and flow experience in technology-aided English learning contexts (Wu and Wang, 2025). Moreover, the extended theory of the TAM model posits that technology acceptance, influenced by external factors, can shape individuals’ learning psychological state and motivation (Cai, 2025; Granić and Marangunić, 2019; Guo and Wang, 2025; Venkatesh and Davis, 2000), which strengthens the theoretical basis for the chained mediation in this research. Hence, the study proposes Hypothesis 4:

Technology acceptance and task flow play a chain mediating role in the relationship between perceived teacher support and EFL learners’ willingness to engage in human-AI collaborative L2 writing.

## 3 Methodology

### 3.1 Context and participants

The participants of this study were EFL learners taking the “College English” course at a university located in central China. In most Chinese universities, this course serves as a mandatory general education course for first-year non-English major students, aiming to cultivate EFL learners’ listening, speaking, reading, and writing skills. To align with current pedagogical and technological developments and address the challenges of the AI era, university administrators advocate English teachers to implement AI-aided teaching reforms to facilitate students’ digital literacy and comprehensive English proficiency. The course teaching team comprised two senior teachers with AI-assisted English teaching experience, one responsible for reading and writing instruction and the other for listening and speaking instruction. The research received approval from the institutional review board, which confirmed that all procedures complied with the Declaration of Helsinki on Educational Research Ethics (Grant number: HNGXY-23-49). The student participants, totaling 530 individuals from six different classes, included 44.7% male learners (237 individuals) and 55.3% female learners (293 individuals), with ages ranging from 18 to 20 years old. Regarding the distribution of majors, 232 students (43.8%) were liberal arts majors, 153 students (28.8%) were science majors, and 145 students (27.4%) were engineering majors. All student participants provided informed consent and took part in the study voluntarily, in strict compliance with local ethical guidelines. They were also assured that their responses to the questionnaire would have no bearing on their academic performance or eligibility for awards and honors.

Although the participants were recruited from several classes, the current study did not adopt a hierarchical linear modeling approach for three practical and methodological reasons. First, the number of classes is relatively small, which does not meet the conventional threshold of at least 30 groups required for stable parameter estimation in hierarchical linear modeling (Maas and Hox, 2005). Second, the participating classes exhibit high similarity in curriculum arrangement, L2 writing teaching mode, and AI-assisted learning arrangements, with the same teacher responsible for the argumentative L2 writing instruction, resulting in negligible between-class variance. Third, all key variables in this study are individual-level psychological perceptual constructs. Given the limited class-level variability and the small number of groups, treating individual observations as the primary unit of analysis is methodologically acceptable in this context.

### 3.2 Research procedure

In the start of the semester in October 2024, the English teacher presented the research details to the six teaching classes and expounded on the utilization of AI-aided L2 writing platform and tools. Every participant was clearly aware of the aim of the study and joined it of their own free will, showing great enthusiasm in cooperation. During the formal teaching period, all six classes followed unified instructional arrangements and identical teaching procedures to ensure consistent teacher support and AI application requirements. The teacher provided ongoing multidimensional support throughout the human-AI collaborative writing process, including procedural guidance on AI tool operation, content strategy instruction for argumentative writing, real-time learning encouragement, and targeted academic feedback. A total of three argumentative writing tasks were assigned at an approximate 1-month interval and adopted classic College English Test Band 4 (CET-4) writing topics. Students were required to complete their English argumentative essays using the Qianyan AI platform, a specialized writing assistant, in comparison with two other types of AI tools: automated writing evaluation systems and large language models such as ChatGPT (Luo et al., 2025). They were guided to explore various collaborative writing functions of Qianyan AI and submit their essays via U Campus, an online platform for the course learning, within the allotted time. Utilizing a blend of AI and manual assessment, the teachers then examined students’ compositions, assigning scores and offering suggestions for enhancement. If students have any questions regarding the use of AI tools or any confusion about human-AI collaborative L2 writing, they could consult the teacher in a timely manner, and targeted explanations and personalized guidance were consistently provided.

The free AI writing assistant is developed and operated by Qianyan Network Technology Co., Ltd., based in Shanghai, China. Its main functional modules include mock exam, intelligent scoring, themed writing, text summarization, expansion, and revision. During this research, the mock exam module was intentionally adopted. Three argumentative writing topics from the CET-4 were chosen, which are: “The Importance of Developing a Healthy Lifestyle Among College Students,” “Are People Becoming Addicted to Technology?” and “The Necessity of Developing Social Skills for College Students.” The platform can generate essay frameworks for students. EFL learners may then independently revise the framework content and compose sentences with the assistance of the built-in large language model. The platform can further recommend words or lexical items and help integrate multiple ideas. Although some AI-generated content may deviate from students’ original intentions, L2 writers retain the autonomy to edit or reject such suggestions at any time. The entire process thus emphasizes the role of humans as decision-makers, highlighting their high-level thinking and executive control abilities in the writing process. This deep collaborative L2 writing model not only helps users correct errors and optimize texts in a timely manner during the writing process, but also encourages users to unleash their thinking potential and improve efficiency when appropriately collaborating with AI.

Following the completion of the final human-AI collaborative writing task in January 2025, a web-based survey was administered via an online questionnaire distributed through the U Campus platform, and students provided their informed consent online. To avoid misunderstandings, all statements in the questionnaire were presented in Chinese. The two teachers independently translated the English scales and then discussed and revised the translations to ensure the accuracy and consistency of the content (Beaton et al., 2000). A preliminary pilot test was conducted with 10 undergraduate students to estimate questionnaire completion time and examine the clarity and comprehensibility of all items. Participants reported that the item wording was clear and the contextual adaptation to human-AI collaborative L2 writing was appropriate. After formal data collection, the first and second authors independently screened all returned questionnaires. Surveys with excessively short completion time and extreme response patterns were excluded. A total of 525 questionnaires were initially collected, among which 22 questionnaires with a completion time shorter than 90 s were eliminated to ensure data quality and research reliability. The final valid sample consisted of 503 usable responses, yielding an effective response rate of 95.80%.

### 3.3 Research instrument

All measurement scales adopted in this study are well-established and widely validated instruments with reliable psychometric properties. No substantial revision, item deletion, or structural adjustment was made to the original scale items. Minor contextual adaptation was conducted only in the situational description to fit the research context of human-AI collaborative L2 writing. After contextual adaptation, the revised questionnaire items were reviewed by several researchers in English teaching and learning. They evaluated the item appropriateness, wording clarity, and contextual relevance to human-AI collaborative writing. Minor revisions were made to ambiguous expressions to ensure adequate content validity.

#### 3.3.1 Perceived teacher support scale

The Chinese version of the perceived teacher support scale, developed and validated by Liu and Li (2023) among senior high school EFL learners in China, was used to assess participants’ perceived teacher support. There are 12 items in the scale, which are used to measure three aspects: academic support, instrumental support, and emotional support. They are arranged on a seven-point Likert scale ranging from “1” (“strongly disagree”) to “7” (“strongly agree”). In the current study, the scale was found to be psychometrically sound, with high validity and reliability (Cronbach’s alphas = 0.970, KMO = 0.961). The factor loadings ranged from 0.820 to 0.915, with CR = 0.975 and AVE = 0.769.

#### 3.3.2 Technology acceptance scale

To assess participants’ technology acceptance during human-AI collaborative L2 writing, we administered a validated questionnaire adapted from Davis’s (1989) Technology Acceptance Model (TAM). The adapted scale was specifically tailored to Qianyan AI, the collaborative writing tool employed in this study. A representative item stated: “I could easily become skilled at using Qianyan tool.” Participants responded to the 12-item scale using a seven-point Likert scale (1 = strongly disagree to 7 = strongly agree). The adapted scale demonstrated strong psychometric properties, with excellent construct validity and high internal consistency (Cronbach’s alpha = 0.937, KMO = 0.941). The factor loadings ranged from 0.876 to 0.938, with CR = 0.980 and AVE = 0.805.

#### 3.3.3 Task flow scale

The foreign language task flow scale, which was developed by Egbert (2003), was used to measure the levels of flow that the participants encountered during the human-AI collaborative L2 wriintg tasks. The original scale was developed for foreign language learning contexts. It has also been validated among Chinese university EFL learners (Cronbach’s alpha = 0.83) in Wang and Huang’s (2022) research, which implies the applicability of the scale in the current study. On a seven-point Likert scale that spans from “1” (“strongly disagree”) to “7” (“strongly agree”), responses are given for the 14 items. In the present study, the scale demonstrated satisfactory psychometric quality, Cronbach’s alpha was 0.797 and the KMO value was 0.932. The factor loadings ranged from 0.484 to 0.897, with CR = 0.946 and AVE = 0.567.

#### 3.3.4 Willing to engage in L2 writing scale

The willingness of EFL learners to engage in human-AI collaborative L2 writing was assessed using the adapted willingness to write scale developed by Rafiee and Abbasian-Naghneh (2020). Some adjustments were made to meet the specific purposes of the present study. A representative item is: “I am eager to establish written communication with foreigners in L2 with the help of AI.” Participants responded to the four items on a seven-point Likert scale, where 1 indicated “strongly disagree” and 7 denoted “strongly agree.” In the present sample, the scale exhibited high reliability and validity, with Cronbach’s alpha = 0.907 and KMO = 0.823. The factor loadings ranged from 0.773 to 0.898, with CR = 0.910 and AVE = 0.718.

## 4 Results

### 4.1 Common method bias test

To mitigate potential common method bias inherent in self-reported questionnaires, several procedural controls were adopted in this study, including guaranteeing participant anonymity and embedding reverse-scored items in selected scales. Furthermore, confirmatory factor analysis (CFA) was conducted to examine common method variance. The results showed that chi-square value of the multi-factor model was χ^2^ = 3224.232, df = 794, which fitted the data significantly better than the single-factor model (△χ^2^ = 5558.815, △ df = 25, *p* < 0.001). This finding suggests that common method bias was not severe in this study (Podsakoff et al., 2003). In addition, the overall measurement model achieved acceptable fit based on Schumacker and Lomax’s (2016) criteria: the chi-square to degrees of freedom ratio is 4.061, SRMR = 0.056, RMSEA = 0.078, NFI = 0.872, IFI = 0.900, TLI = 0.891, CFI = 0.900, PNFI = 0.804, PCFI = 0.830.

Nevertheless, given the relatively high correlations among key study variables and the cross-sectional self-report design, we acknowledge that slight inflation of relational strength caused by shared method variance cannot be completely ruled out. Therefore, the associations observed in this study should be interpreted with appropriate caution.

### 4.2 Descriptive statistics and correlation analysis

The means, standard deviations, and correlation coefficients among the study variables are presented in Table 1. Data shows that the mean score for university EFL learners’ task flow experience is 4.450, indicating that the participants generate moderate flow in the human-AI collaborative L2 writing tasks (Egbert, 2003). Such moderate flow may act as a buffer against over-immersion in AI-generated content, balancing cognitive engagement with critical evaluation and fostering effective, reflective human-AI collaboration. The mean scores for perceived teacher support, technology acceptance, and willingness to engage in human-AI collaborative writing are all above 5, suggesting that the surveyed EFL learners’ perceived teacher support and AI technology acceptance are at a moderately high level, and they have a moderately strong willingness to engage in the specific writing tasks. Furthermore, the results of the correlation analysis show that the correlation coefficients among the variables range from 0.587 to 0.779, indicating moderate to high positive correlations, all of which reach the level of significance. The correlation coefficients of all variables are below the square root of their respective AVEs, indicating discriminant validity. Given the relatively high correlation coefficients, we further examined the data using SmartPLS. The Heterotrait-Monotrait Ratio (HTMT) report shows that the cross-loadings of all variables range from 0.780 to 0.858, which is below the threshold of 0.90. Thus, the discriminant validity between constructs falls within an acceptable range (Henseler et al., 2015). Thus the study variables meet the basic requirements for hypothesis testing in the regression model (Hair et al., 2019).

**TABLE 1:** Descriptive statistics and correlation analysis of variables.

Item	Mean	Standard deviation	Perceived teacher support	Technology acceptance	Task flow	Willingness to engage
Perceived teacher support	5.420	1.196	0.876	0.807	0.800	0.830
Technology acceptance	5.232	1.139	0.767***	0.897	0.780	0.819
Task flow	4.450	0.613	0.634***	0.587***	0.752	0.858
Willingness to engage	5.285	1.230	0.779***	0.763***	0.641***	0.847

***P < 0.001. Diagonal values are square roots of AVE. Above-diagonal values are HTMT cross-loadings. Below-diagonal values are correlation coefficients.

### 4.3 Hypothesis test and mediation pathway analysis

In this study, Model 4 and Model 6 in the PROCESS macro developed by Andrew F. Hayes were used to test the mediating pathways of technology acceptance and task flow on the relationship between perceived teacher support and WTE in human-AI collaborative L2 writing, with the above methodological justification for not further modeling the nested class structure. The bias-corrected percentile bootstrap method was used to resample data and validate each model. If the 95% confidence interval does not include 0, the path effect is considered statistically significant at the *p* < 0.05 level (Shrout and Bolger, 2002).

The analysis of the mediation role of technology acceptance is shown in Table 2. The results show that perceived teacher support among EFL learners is directly and positively associated with WTE (β = 0.801, *p* < 0.001), supporting Hypothesis 1. Perceived teacher support is also directly and positively associated with technology acceptance (β = 0.730, *p* < 0.001). When perceived teacher support and technology acceptance are examined simultaneously in relation to WTE, the positive correlations of perceived teacher support (β = 0.484, *p* < 0.001) and technology acceptance (β = 0.434, *p* < 0.001) with WTE are significant, with a decrease compared to the total effect. As the 95% confidence interval of the model does not contain 0, it shows that the partial mediating role of technology acceptance is verified. Consequently, Hypothesis 2 is proven to be valid.

**TABLE 2:** Mediation effect test of technology acceptance.

Item	WTE		Technology acceptance		WTE	
	β	95% CI	β	95% CI	β	95% CI
Perceived teacher support	0.801***	0.745; 0.858	0.730***	0.677;0.784	0.484***	0.403; 0.564
Technology acceptance	–	–	–	–	0.434***	0.349; 0.518
R^2^	0.779		0.766		0.820	
F	773.60		715.37		515.78	

***P < 0.001.

As presented in Table 3, the mediation analysis revealed that perceived teacher support among Chinese EFL learners was significantly and positively correlated with task flow (β = 0.325, *p* < 0.001). When perceived teacher support and task flow experience were entered simultaneously in relation to L2 writing willingness, both perceived teacher support (β = 0.641, *p* < 0.001) and task flow experience (β = 0.491, *p* < 0.001) show significant positive associations with WTE. The values of the coefficients are lower than the overall effect, and the 95% confidence interval of the model does not contain 0, which suggests that the partial mediating role of task flow experience is validated. Consequently, Hypothesis 3 is verified.

**TABLE 3:** Mediation effect test of task flow.

Item	WTE		Task flow		WTE	
	β	95% CI	β	95% CI	β	95% CI
Perceived teacher support	0.801***	0.745; 0.858	0.325***	0.290; 0.359	0.641***	0.571; 0.711
Task flow	–	–	–	–	0.491***	0.355; 0.628
R^2^	0.779		0.634		0.801	
F	773.60		337.24		449.83	

***P < 0.001.

To further verify the chain mediation role of technology acceptance and task flow on the relationship between EFL learners’ perceived teacher support and WTE in human-AI collaborative writing, Model 6 within the PROCESS macro was used. The original sample was used as the bootstrap population, with 2000 resamples and a 95% confidence interval for the mediating effect. According to Shrout and Bolger (2002), when the 95% confidence intervals of the path coefficients do not contain 0, it means that the chain mediation effect is significant. Table 4 presents the total effect, direct effect, and the indirect effect values for the three indirect paths, along with their 95% confidence intervals to test significance. The results show that the chain mediating role is statistically significant (β = 0.035, 95% CI [0.013, 0.063], which excludes 0), accounting for 8.77% of the total effect. Among the three indirect paths, the mediating pathway of technology acceptance is the strongest, with an effect size of.281, accounting for 70.42% of the total effect. Thus, Hypothesis 4 was supported.

**TABLE 4:** Path and effect decomposition of perceived teacher support on WTE.

Effect type	Effect size	95% CI lower	95% CI upper	Effect proportion (%)
Total effect	0.801	0.745	0.858	–
Direct effect	0.399	0.315	0.482	–
Ind 1: PTS→TA→WTE	0.281	0.194	0.365	70.42
Ind 2: PTS→TF→WTE	0.085	0.044	0.132	21.30
Ind 3: PTS→TA→TF→WTE	0.035	0.013	0.063	8.77

PTS, perceived teacher support; TA, technology acceptance; TF, task flow; WTE, willingness to engage in human-AI collaborative L2 writing.

## 5 Discussion

### 5.1 Associations between perceived teacher support and EFL learners’ WTE in human-AI collaborative L2 writing

The research findings indicate that perceived teacher support is directly and positively linked to EFL learners’ willingness to engage in human-AI collaborative L2 writing. This finding highlights the crucial role of perceived teacher support in EFL learners’ willingness to engage in AI-related writing tasks, which is consistent with the research of Yu et al. (2024) and further validates the importance of teacher support within the framework of AI-aided language learning (Lee and Lee, 2024). It also confirms that despite the rapid advancement of AI in education, AI cannot fully replace the role of human foreign language teachers, particularly in fostering emotional connection and empathetic engagement (Seyri and Ghiasvand, 2025; Yang, 2024). These findings further suggest that a supportive language learning environment, combined with AI-aided learning opportunities, may contribute to increased learners’ willingness to engage in collaborative L2 writing. Moreover, guidance on the appropriate and responsible use of AI tools appears crucial in preventing excessive reliance on AI-generated content. Such instructional practices may in turn promote learners’ initiative and active participation in human-AI collaborative L2 writing tasks. It is also noteworthy that the correlation between perceived teacher support and willingness to engage is relatively high. While these strong associations are consistent with theoretical expectations, they require cautious interpretation. First, the high correlations may partially reflect conceptual overlap across constructs. For instance, perceived teacher support in L2 writing contexts inherently contains motivational and affective support that can directly promote students’ willingness to take part in human-AI L2 writing activities. Second, all variables were measured via self-reported questionnaires at a single time point. Although a common method bias test was performed, such potential confounding effects cannot be completely ruled out.

### 5.2 The independent mediating roles of technology acceptance and task flow

The study revealed two independent mediating paths through which perceived teacher support associates with EFL learners’ WTE in human-AI collaborative L2 writing. First, technology acceptance emerged as a significant mediator, which is consistent with the research by Deng et al. (2026). This finding also aligns with the extended TAM framework, wherein teacher support operates as a social relational factor that enhances learners’ intentions to adopt technology through dual pathways: establishing subjective norms via mandatory requirements and fostering voluntary compliance through demonstrated utility (Venkatesh and Davis, 2000). Learners with higher levels of perceived teacher support tend to hold more positive attitudes toward AI writing assistants, which is closely linked to their perceived usefulness and ease of use of such technologies (Liang et al., 2026). As learners recognize that AI tools can effectively improve the quality, accuracy, and efficiency of their L2 writing, their willingness to engage in AI-supported writing activities increases accordingly. Previous research has raised concerns that AI tools may lead to learners’ over-reliance and potentially trigger metacognitive laziness, a tendency in which learners passively offload thinking and monitoring to AI rather than engaging in active, self-regulated learning (Fan et al., 2024). This suggests that while technology acceptance is crucial for initiating students’ interest and intention to participate in AI-aided learning, it does not necessarily guarantee their full and effective engagement in the learning process.

Secondly, the research discovered the independent mediating role of task flow during the process in which perceived teacher support relates to EFL learners’ WTE. This finding is consistent with earlier studies that regarded flow experience as a mediator between contextual factors and language learning outcomes (Li et al., 2021; Liu and Song, 2021; Wu and Wang, 2025). Task flow, as a situational psychological state, can stimulate EFL learners’ intrinsic motivation, enjoyment, and satisfaction in specific language tasks (Cai, 2025; Dewaele et al., 2023), and this in turn exerts positive influences on their language learning achievements. It stands to reason that EFL learners experiencing a state of flow are more prone to attaining enhanced learning satisfaction when leveraging AI tools for collaborative writing, so as to enhance their willingness to engage in AI-aided collaborative L2 writing, which is positively associated with deeper cognitive investment in learning. Notably, the moderate level of task flow observed in this study not only boosts WTE but also serves as a buffer against excessive immersion in AI-generated content without critical evaluation, thus supporting balanced and reflective human-AI collaboration.

### 5.3 The chained mediating role of technology acceptance and task flow

This study demonstrates that technology acceptance has a facilitative role on EFL learners’ task flow experience in human-AI collaborative writing L2 process. The theoretically hypothesized chain mediating pathway linking perceived teacher support to WTE via technology acceptance and task flow is statistically significant. This finding further validates the S-O-R framework by revealing the associative relational pathway among external contextual factors, individual psychological states, and behavioral intention, rather than confirming a strict temporal progression. In essence, technology acceptance is a cognitive and affective state that affects learners’ motivation and acceptance of learning with technology (Granić and Marangunić, 2019; Xu et al., 2024). EFL learners with higher perceived usefulness and acceptance of AI writing tools are more willing to integrate such technology into L2 learning. With technological assistance, learners can access more academic resources and further unleash their learning potential, thereby reducing L2 writing anxiety and enhancing metalinguistic awareness through reflecting on linguistic choices prompted by AI-generated suggestions (Luo et al., 2025). Positive technology acceptance is closely associated with optimal psychological engagement in learning, which is characterized by clear goal orientation and a balanced match between task challenges and personal skills, creating favorable conditions for the emergence of task flow (Egbert, 2003). These flow experiences appear to be more common in collaborative writing, because the completion of the task induces greater intrinsic motivation and calls for increased concern and control over one’s role in the process of essay content negotiation (Ghanbaran et al., 2023; Li et al., 2026). Accordingly, within the specific context of human-AI collaborative L2 writing, the interactive writing process with AI further facilitates the emergence of a flow state. Notably, the role of teachers is even more crucial in AI-aided foreign language learning contexts (Yang, 2024). Teachers can provide necessary instrumental support, such as AI-assisted writing tools and guidance on academic integrity, as well as emotional support including patience and language learning encouragement. In addition, they are also obligated to offer academic support, such as instruction in grammar and the construction of argumentative essays. This enables students to appropriately evaluate and revise the collaboratively-generated essays and ensures that the L2 writing process is shielded from the negative impacts of changes in the external technological environment.

### 5.4 Limitations and future directions

Although this research has made some contributions, it also has some limitations. First, this study adopted a cross-sectional design, with all variables measured simultaneously at a single time point. Such a research design limits rigorous inferences regarding the temporal sequence of variables and definitive causal relationships. Accordingly, the chain mediating pathway identified in this study should be interpreted only as theoretically hypothesized statistical associations, rather than a confirmed sequential causal process. Moreover, all data were collected via self-report questionnaires at a single time point. The relatively high correlations among key variables may be partially attributed to shared method variance, even though procedural remedies and CFA-based common method bias testing were implemented. Such potential method effect may slightly inflate the observed relationships among constructs, which warrants cautious interpretation of the effect sizes and correlation magnitudes. Future research could address these limitations by adopting longitudinal designs to explicitly validate the temporal order of the chain mediating pathway, employing multi-source data collection, or refining questionnaire items to yield more robust and generalizable findings. Furthermore, the study measured EFL learners’ willingness to engage, which is relatively easy to assess through self-reporting. However, evaluating students’ actual engagement could be a more critical research focus in the AI era. Future studies may explore students’ actual engagement across various aspects such as cognitive, emotional, and behavioral aspects. This could be achieved by making use of multi-modal data sources like electroencephalogram (EEG) and eye-tracking experiments (Barrett and Pack, 2023). In addition, the present study focused on learners’ willingness to engage in human-AI collaborative L2 writing tasks, as this willingness has been viewed as positively contributing to learners’ L2 performance and foreign language enjoyment (Zhang and Zhang, 2024). However, the specific impacts of human-AI collaboration on L2 writing outcomes, as well as the extent to which it may enhance L2 writers’ higher-order cognitive skills, remain underexplored and warrant further investigation. Therefore, future research could examine the quality of writing produced in human-AI collaborative contexts and explore how pedagogical practices of human-AI collaborative L2 writing may influence EFL learners’ linguistic development and cognitive growth. Finally, even though we have provided methodological justification for not adopting hierarchical modeling, the nested data structure of students within classes cannot be completely ignored. Future studies with a larger number of classes and a more rigorous stratified sampling design may adopt multi-level models to further verify the current findings and account for potential clustering effects.

## 6 Conclusion and implications

Through descriptive analysis and regression analysis, the present research investigated the association between teacher support and Chinese university EFL learners’ willingness to engage in human-AI collaborative writing. The results show that perceived teacher support is directly and positively correlated with EFL learners’ WTE. In addition to the direct relational association, the research identified indirect relational links via two separate mediators, technology acceptance and task flow, along with a chain-mediating pathway that includes both. The results confirm the comprehensive theoretical framework combining TAM and the S-O-R model. This framework highlights how external support and EFL learners’ cognitive-affective states contribute to their intention to engage in technology-enhanced language learning. Furthermore, the results offer significant insights that can direct future AI-aided L2 writing instruction, introducing innovative approaches for its implementation in the age of AI.

The findings of this study may yield several implications for future research and EFL pedagogical practices. Theoretically, this study contributes to the growing body of literature on perceived teacher support and WTE in L2 writing contexts by investigating its dynamic interplay with AI technologies. In addition, the integrated theoretical framework of TAM and S-O-R model has been empirically verified in this study, therefore extending the scope of application of these two theories in the field of L2 writing instruction. This provides a solid theoretical foundation for future research, which can further explore additional factors that shape learners’ engagement willingness, such as self-efficacy, academic anxiety, and attitudes toward technology, thereby expanding and refining the integrated model (Chahal and Rani, 2022; Chen et al., 2024; Zhang and Jiang, 2025). Incorporating a greater number of variables enables a more thorough comprehension of the behavioral intentions of EFL learners within AI-aided language learning settings.

Pedagogically, the findings can inform EFL teachers on how to effectively leverage teacher support to enhance students’ willingness to engage in human-AI collaborative L2 writing. A multifaceted support system, including technical guidance, writing strategies instruction, emotional support, and timely feedback, can be proactively implemented to foster positive learner experiences. The study further highlights the mediating role of EFL learners’ cognitive and affective states in human-AI collaborative L2 writing. Accordingly, the design of AI-assisted writing tasks needs to take into account not only the perceived ease of use and usefulness of the technology itself but also learners’ emotional experiences such as task flow (Seyri and Ghiasvand, 2025; Wu and Wang, 2025). Educators thus should integrate AI tools into L2 writing instruction through collaborative learning approaches, demonstrating concrete applications and implementation strategies to enhance students’ perceptions of the technology’s usefulness and ease of use. Systematic supervision can also be provided throughout students’ human-AI collaborative writing processes. While monitoring their writing progress and the content generated by AI, instructors may prioritize the cultivation of students’ technological proficiency and academic integrity, so as to prevent excessive dependence on AI and ensure the effective and responsible use of such tools (Yang and Lin, 2025). Furthermore, follow-up translation tasks or cross-cultural comparison activities based on students’ initial L2 writing outputs should be designed to foster more flow experiences through diversified language activities. This, in turn, can strengthen students’ willingness to engage in human-AI collaborative writing and contribute to the production of high-quality written texts.

It should also be emphasized that the effective implementation of human-AI collaborative L2 writing instruction relies on the joint efforts of teachers, students, and ongoing technological advancement. On the one hand, AI writing assistants should be not only user-friendly and engaging but also capable of adapting to the diverse needs and preferences of individual learners. On the other hand, teachers can actively cultivate learners’ critical thinking to avoid blind reliance on AI, and EFL learners should learn to appropriately integrate AI-generated content while preserving their own ideas and authentic voice in writing. Only after these requirements are satisfied can this cooperative writing model truly realize its potential and become the innovative direction that L2 experts have been longing for.

## Data Availability

The raw data supporting the conclusions of this article will be made available by the authors, without undue reservation.
